# Hydration Status Is Associated with Aortic Stiffness, but Not with Peripheral Arterial Stiffness, in Chronically Hemodialysed Patients

**DOI:** 10.1155/2015/628654

**Published:** 2015-06-17

**Authors:** Daniel Bia, Cintia Galli, Rodolfo Valtuille, Yanina Zócalo, Sandra A. Wray, Ricardo L. Armentano, Edmundo I. Cabrera Fischer

**Affiliations:** ^1^Physiology Department, School of Medicine, CUiiDARTE, Republic University, 11800 Montevideo, Uruguay; ^2^National Council of Technical and Scientific Research (CONICET), C1033AAJ Buenos Aires, Argentina; ^3^Technological National University, C1179AAQ Buenos Aires, Argentina; ^4^Fresenius FME Burzaco, B1852FZD Buenos Aires, Argentina; ^5^Favaloro University, Solis, C1093AAS Buenos Aires, Argentina

## Abstract

*Background*. Adequate fluid management could be essential to minimize high arterial stiffness observed in chronically hemodialyzed patients (CHP). *Aim*. To determine the association between body fluid status and central and peripheral arterial stiffness levels. *Methods*. Arterial stiffness was assessed in 65 CHP by measuring the pulse wave velocity (PWV) in a central arterial pathway (carotid-femoral) and in a peripheral pathway (carotid-brachial). A blood pressure-independent regional arterial stiffness index was calculated using PWV. Volume status was assessed by whole-body multiple-frequency bioimpedance. Patients were first observed as an entire group and then divided into three different fluid status-related groups: normal, overhydration, and dehydration groups. *Results*. Only carotid-femoral stiffness was positively associated (*P* < 0.05) with the hydration status evaluated through extracellular/intracellular fluid, extracellular/Total Body Fluid, and absolute and relative overhydration. *Conclusion*. Volume status and overload are associated with central, but not peripheral, arterial stiffness levels with independence of the blood pressure level, in CHP.

## 1. Introduction

The increased mortality observed in chronic kidney disease compelled analyzing the role of the traditional risk factors and those derived from recent studies that are particularly relevant in chronically hemodialyzed patients (CHP). Consequently, left ventricular hypertrophy, malnutrition, and hydration status (i.e., overhydration, OH) in end stage renal disease have become “novel risk factors” [1–4]. Volume overload is considered a predictor of outcome in CHP [5], in which systemic hypertension is also well documented; however OH is not always accompanied by volume-dependent high blood pressure (BP). Previous reports demonstrated that hypertension is not always fluid-dependent in CHP [6]. Consequently, at present, a Normal Hydration State is a very important target to be taken into account during renal replacement therapy.

At least in theory, an adequate fluid management could be essential to minimize the high arterial stiffness observed in CHP. Even so, it is not still clear how volume overload affects the arterial system and the nature of the association with other novel risk factors [7]. Recently, Hur et al. have reported that fluid evaluation with bioimpedance spectroscopy (BIS) determines an improvement in the management of CHP and decreases in arterial stiffness [8]. However, the mentioned authors were incapable of determining if the origin of the arterial stiffness improvement was due to volume overload [8]. In other words, whether volume overload determines a BP dependent or independent arterial stiffness increase and whether these potential effects are similar in central (i.e., elastic) and peripheral (i.e., muscular) arteries remain to be analyzed.

From a different point of view, cardiovascular disease is a well-known leading cause of the increased mortality observed in CHP and is associated with aortic stiffening [9, 10]. More than a decade ago, Blacher et al. demonstrated that the measurement of arterial pulse wave velocity (PWV; “gold standard” parameter to measure regional arterial stiffness) has prognostic power to predict mortality in end stage renal disease [9]. However, the physiopathological mechanisms that determine the increased risk have not been properly elucidated.

At present, the hydration status of the human body can be assessed by multifrequency bioimpedance [11]. Furthermore, using BIS and applying a 2-compartment (2-C) model of body composition (BC; fat-free mass and fat mass), a significant association between volume overload (evaluated as extracellular [ECF] to intracellular water [ICF] ratio) and aortic stiffness was reported [7, 12]. Nevertheless, at least four aspects of this association should be noted: (a) Is the volume overload-arterial stiffness relationship the same in elastic and muscular arteries? (b) Does the mentioned association persist when the hydration status is quantified using another model, such as the three compartment (3-C) model? (c) Does this hydration status-arterial stiffness association persist if vascular stiffness is obtained with independence of the BP levels of the patient? (d) Is the association similar when a comparison between right and left arterial territories of the human body is performed?

In this context, the aim of this study was to determine the hydration status using a 3-C model and the central (i.e., elastic artery) and peripheral (i.e., muscular artery) arterial stiffness levels in CHP, in order to evaluate the potential BP dependent and/or independent association between body fluid status (OH, OH/ECF, ECF/ICF, and ECF/Total Body Fluid (TBF)) and central (carotid-femoral pathway) and peripheral (carotid-brachial) arterial stiffness levels.

## 2. Methods

This research was carried out in a single health care institution (Fresenius Medical Care FME Burzaco, Buenos Aires, Argentina). From an initial cohort of ambulatory CHP (*n* = 104), sixty-five patients were enrolled in this cross-sectional research. The inclusion criteria were as follows: (1) patients are on hemodialysis for more than 3 months, (2) the vascular access is placed in the upper limb, and (3) patients have had no acute cardiovascular events in the last 3 months. The exclusion criteria were as follows: (1) patients abusing alcohol, (2) patients with lower or upper extremity amputation, (3) patients with symptomatic carotid artery stenosis, (4) patients with uncontrolled diabetes mellitus, (5) patients unwilling to participate in the investigation, (6) patients with cardiac arrhythmias, and (7) patients with metallic implants (stents, pacemakers, etc.). Our Institutional Review Board and Ethics Committee approved this study. All patients gave their written consent to participate in the study.

### 2.1. Measurements

Before their midweek hemodialysis session, patients were subjected to the measurement of PWV and of other physical parameters, such as arterial BP, body weight, standing height, and waist and hip perimeter. The hydration status was determined through a bioimpedance study. Blood was drawn from each patient and routine chemical analyses were performed to quantify haematocrit, haemoglobin, serum creatinine, calcium, phosphate, serum albumin, parathyroid hormone (PTH), urea, total cholesterol, HDL and LDL cholesterol, and triglyceride.

In all patients, brachial BP was measured in the contralateral upper limb to which the functioning vascular access was confectioned. The patients were allowed to rest in supine position during 15 minutes before the BP measurement. Pressure was determined using a digital automatic BP monitor (Omron model HEM 781 INT). Heart rate, waist circumference, and hip perimeter were also measured, and the body mass index (BMI) was calculated by dividing weight by height squared.

The study of body fluids was done using a bioimpedance monitor (Fresenius Medical Care, (BCM) Body Composition Monitor OP-ES, software version: 3.2.x edition: 8/03.12). The BCM is a BIS device that measures 50 different frequencies that range from 5 to 1000 kHz, analyzing the whole-body bioimpedance. The BCM discriminates fluid of the intra- and extracellular water content of lean tissue mass (LTM), adipose tissue mass (ATM), and excess fluid (OH). LTM, ATM, and OH are obtained from measurements of body weight, height, and whole-body ICF and ECF determined by BIS. The body composition model determines whether changes in ICF and ECF reflect an increase or a loss of ATM or LTM. This is the only device that identifies OH as a third compartment on the basis of a unique body composition model. OH represents the excess fluid (fluid overload) stored almost exclusively in the extracellular volume of a patient and is therefore part of the ECF, whereas the water of LTM and ATM consists of differing proportions of ECF and ICF, in addition to solid components. Applying the 3-C model, the following parameters were recorded in each patient: Lean Tissue Index (LTI (kg/m^2^)); Fat Tissue Index (FTI (kg/m^2^)); Total Body Fluid (TBF (L)); ECF (L); ICF (L); and OH (L). Three volume ratios were quantified: ECF/ICF, ECF/TBF, and OH/ECF. Over and subhydration were defined when the OH/ECF ratio was >15% or <0%, respectively [4, 6].

Measurements of PWV were performed on the right and left carotid-femoral and carotid-brachial pathways, using a previously validated instrument (Arteriometer V100, OxyTech, Buenos Aires, Argentina), according to the technique previously and widely described [13–15]. In order to evaluate the aortic and upper limb regional arterial stiffness by means of PWV, the carotid and femoral (or brachial) pulse waves were recorded using mechanotransducers, simultaneously placed on the skin over the carotid and femoral (or brachial) arteries, with the subjects in supine position. Once adequate pulse waveforms were recorded, the time delay between the waveforms (pulse transit time) was measured. To this end, the well-known algorithm to detect the pulse waveform “foot,” called the maximal systolic upstroke algorithm, was used [15]. The distance between the carotid and the femoral (or brachial) sites was used together with the pulse transit time to calculate PWV. The reported value of PWV for each subject was the average of at least 8 consecutive beats automatically calculated. Before calculating PWV, brachial pressure and heart rate were recorded.

PWV is essentially dependent on BP. Studies have shown a significant association between PWV and systolic BP in CHP. To overcome this disadvantage, a BP-normalized stiffness index (*β*) was recently developed and proposed as a new parameter for evaluating regional arterial stiffness, independently of BP level [16]. The equation to quantify *β* is derived from the Bramwell-Hill equation and the stiffness parameter *β* may be less influenced by BP than PWV, and its measurement has been shown to be reproducible. We quantify *β* as *β* = Ln(SBP/DBP) · (2 · *ρ* · PWV^2^/PP), where Ln is the natural logarithm and SBP, PP, and DBP are the systolic, pulse, and diastolic BP, respectively [16].

### 2.2. Statistical Analysis

Continuous variables are expressed as mean value ± standard deviation. ANOVA (Bonferroni test) and *χ* ^2^ analysis were used to analyze differences among the normal hydration state (NHS) group, the overhydration (OH) group, and the subhydration (SH) group. Pearson's correlation was run to determine the relationship between PWV (or *β*) and volume status parameters (ECF/ICF, ECF/TBF, OH, andOH/ECF). All analyses were completed with SPSS software, version 20.0 (SPSS, Chicago, IL, USA). A *P* value of <0.05 was regarded as statistically significant.

## 3. Results

Data collection was successful for all patients included in this study. In all patients, arterial BP and the hydration status were routinely monitored allowing, when necessary, the fluid correction during the renal replacement therapy session and/or the use of pharmacological resources.

Table 1 shows the anthropometric, hemodynamic, and blood characteristics for the entire analyzed population and the three hydric state-related groups. There were no differences in age among groups and in BP levels between the normal and OH group.

Table 2 shows the body water parameters quantified for the entire population and the three hydric state-related groups. Note that the net levels of ECF, ICF, and TBF were not different among groups, but the relative distribution of the water in the extracellular and intracellular components was different, determining differences in ECF/ICF, ECF/TBF, OH, and OH/TBF.

Table 3 shows the arterial stiffness levels for the entire group and the three hydric state-related groups. The OH group showed higher carotid-femoral (but not carotid-brachial) arterial stiffness level than the normal hydric state group (*P* < 0.05). Additionally, note that differences in carotid-femoral arterial stiffness between OH and dehydration groups disappeared when a BP-independent stiffness index (*β*) was calculated. Additionally, there were no differences in carotid-brachial arterial stiffness among groups, with independence of the employed parameter (PWV or *β*).

**Figure 1:**
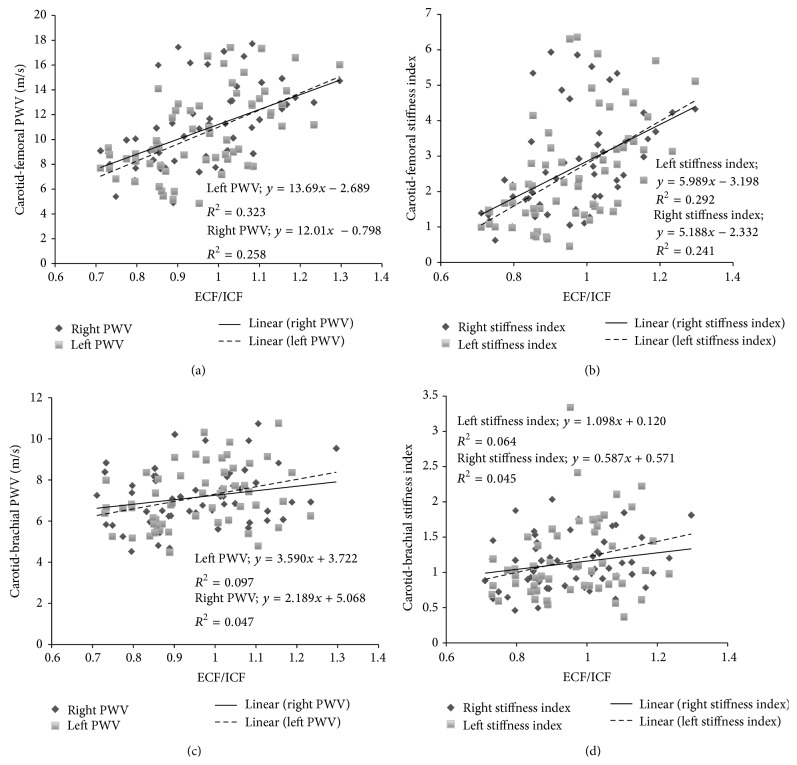
Pulse wave velocity (PWV) and hydration status evaluated through extracellular/intracellular fluid ratio (ECF/ICF) measured using a multi-impedancimetric technique. The carotid-femoral PWV-ECF/ICF relationship shows a significant relationship (*P* < 0.05), as does the stiffness index (*β*)-ECF/ICF relationship (*P* < 0.05).

The carotid-femoral PWV was positively correlated (*P* < 0.05) with the extracellular-to-intracellular fluid volume ratio (ECF/ICF), as seen in Figure 1(a). Our analysis shows the same results, for carotid-femoral PWV obtained both for the left and right body side. Furthermore, the mentioned association has shown to be independent of the BP level as it is when calculating *β* (Figure 1(b)). However, these results could not be confirmed when the right and left aorto-axilo-humeral pathway was considered and evaluated using PWV and *β* (Figures 1(c) and 1(d)).

**Figure 2:**
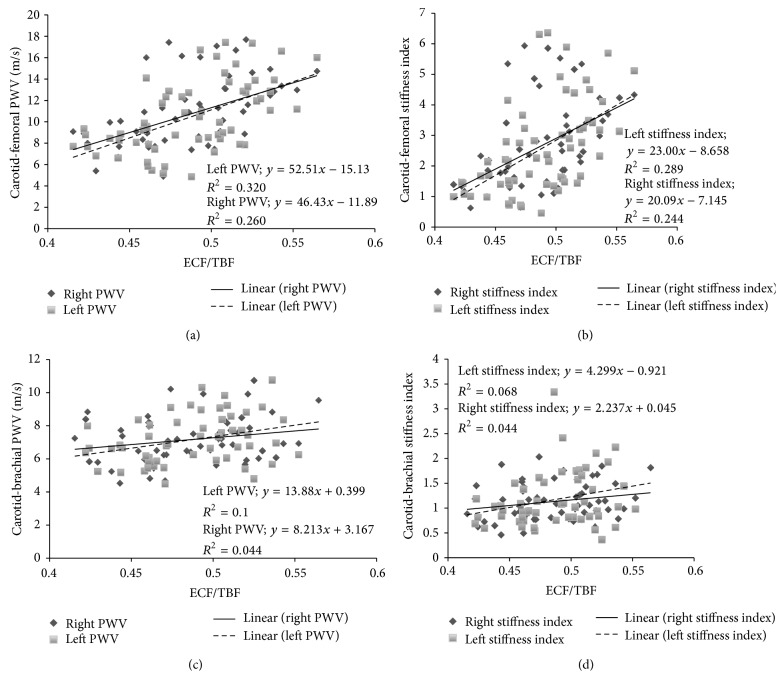
Pulse wave velocity (PWV) and hydration status evaluated through extracellular/Total Body Fluid ratio (ECF/TBF) measured using multi-impedancimetric technique. The carotid-femoral PWV-ECF/TBF relationship shows a significant relationship (*P* < 0.05), as the stiffness index (*β*)-ECF/TBF relationship (*P* < 0.05).

The ECF/TBF was found to be associated with the PWV value in the carotid-femoral pathway, but not in the aorto-axilo-humeral pathway. As seen in Figure 2(a), a positive correlation (*P* < 0.05) was found in both the left and the right PWV carotid-femoral pathways. Moreover, the *β*-ECF/TBF relationship (*P* < 0.05) shows that the mentioned changes are independent of the BP level (Figure 2(b)). Nevertheless, the right and left carotid-brachial pathway *β* level were not correlated with the ECF/TBF ratio (Figures 2(c) and 2(d)).

**Figure 3:**
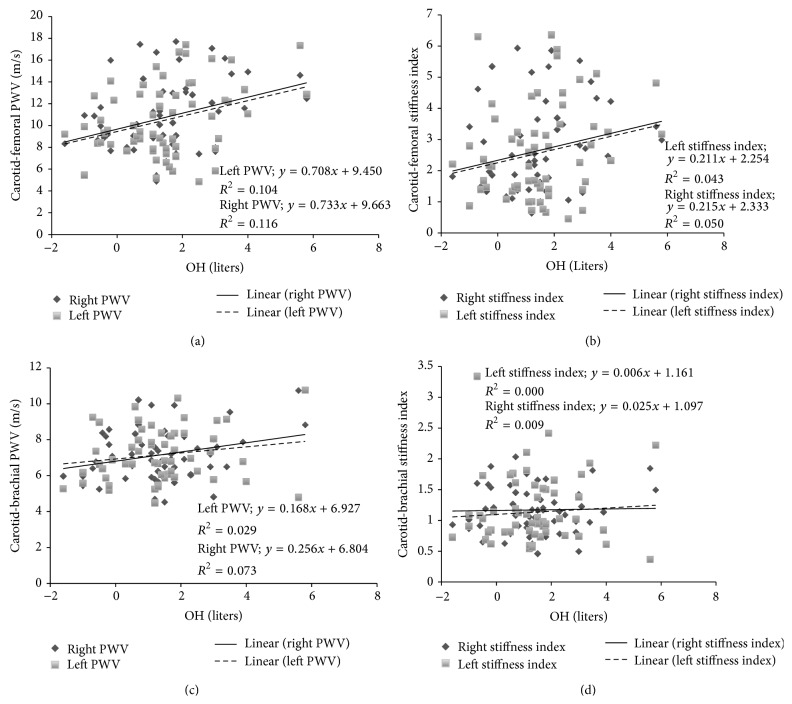
Pulse wave velocity (PWV) and hydration status evaluated through extracellular/overhydration ratio (OH) measured in absolute values using the multi-impedancimetric technique. The carotid-femoral PWV-OH relationship shows a significant relationship (*P* < 0.05), as does the stiffness index (*β*)-OH relationship (*P* < 0.05).

**Figure 4:**
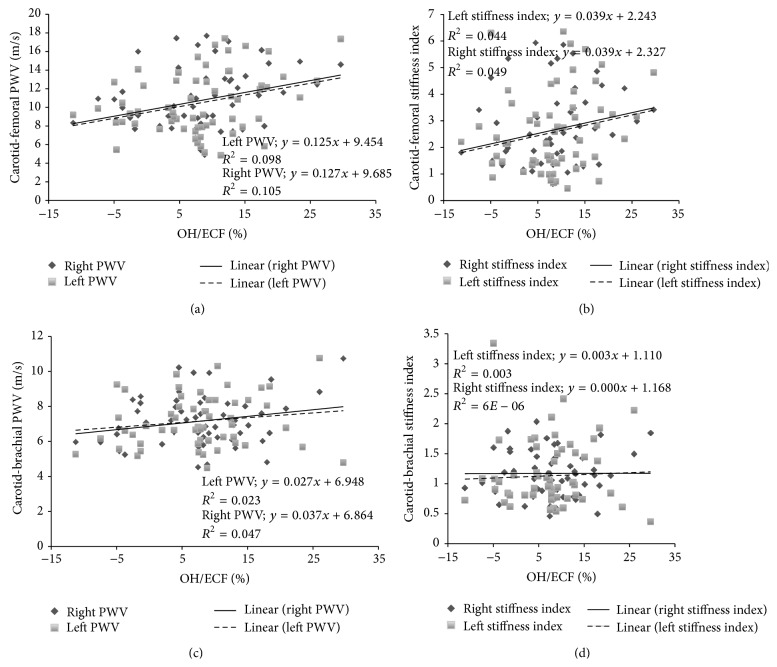
Pulse wave velocity (PWV) and hydration status evaluated through overhydration/Extracellular fluid ratio (OH/ECF) measured in relative values using multi-impedancimetric technique. The carotid-femoral PWV-OH/ECF relationship shows a significant relationship (*P* < 0.05), as does the stiffness index (*β*)-OH/ECF relationship (*P* < 0.005).

Overhydration (OH) measured in absolute units (liters) showed a low (but significant) correlation (*P* < 0.05) with the right and left carotid-femoral PWV (Figure 3(a)). This positive relationship was not confirmed when *β* value was used, not in the right carotid-femoral pathway, or in the left one. Additionally, the right and left carotid-humeral PWV did not show correlation with OH. Finally, when OH was quantified in relative terms (%), the right and left arterial stiffness OH/ECF relationship, analyzed in the carotid-femoral and carotid-brachial pathway, showed similar results that those obtained using absolute values (see Figure 4).

## 4. Discussion

In this work, the relationship between the hydration status and arterial stiffness is analyzed using, for the first time, a modern device that measures 50 different frequencies (from 5 to 1000 kHz), in order to characterize the whole-body bioimpedance. This technology allows using a 3-C model that ensures a reliable quantification of the body fluid compartment. According to the aim of this research, we characterized the arterial stiffness (1) of elastic and muscular arteries and (2) in both the right and left arterial territories of the patients. These approaches allow us to conclude the following:

1. Fluid overload, quantified in terms of absolute values (OH) and of relative estimations (OH/ECF) through BCM, showed a significant BP dependent association with aortic stiffness, but not with peripheral arterial stiffness evaluated in the carotid-brachial pathway.
2. Extracellular fluid relative increases (ECF/ICF and ECF/TBF) were significantly associated with aortic stiffness and with independence of the BP levels. This relationship was not observed when peripheral arterial stiffness was considered. This is an important, original finding [7, 12], which proves that the association of aortic stiffness and hydration status should not be extrapolated to other arterial territories, such as the upper limbs.
3. Fluid overload patients showed significant higher levels of arterial stiffness, evaluated through carotid-femoral PWV, with respect to those that exhibit normal or low hydration status. This difference was not observed when arterial stiffness was analyzed in terms of the *β* index, indicating that the observed differences were BP dependent (Table 3). On the contrary, the analysis in terms of peripheral arterial stiffness showed nonsignificant differences between overhydrated and normally hydrated patients, with independence of blood pressure levels.
4. Dehydratated patients showed nonsignificant differences in terms of arterial stiffness with respect to hemodialyzed patients with normal hydration status, both in terms of aortic and in terms of peripheral stiffness (Table 3).

Our results showed that ECF/ICF and ECF/TBF ratios are associated with carotid-femoral PWV, with independence of BP, but not with carotid-brachial PWV (Figure 1). These findings are partially coincident with those previously reported by Lin et al. [12] and Zheng et al. [7]. In the mentioned reports, the carotid-femoral PWV was correlated with ECF/ICF [12] and ECF/TBF [7], respectively. However, the authors omitted mentioning if such association persists when arterial stiffness is analyzed with independence of arterial BP. Moreover, Lin et al. and Zheng et al. results could not be confirmed when the aorto-axilo-humeral pathway was considered and evaluated using carotid-brachial PWV. Therefore, our results indicate (for the first time) that the increase in the ECF/ICF or ECF/TBF ratio did not provoke a similar change in the arterial stiffness of different arterial pathways. In other words, to affirm that ECF/ICF or ECF/TBF ratio is associated with the arterial stiffness increase in large arteries, as previously mentioned [12], is an incorrect generalization. The research reported by Lin et al. and Zheng et al. was carried out using a similar bioimpedance technique but not the same one. Consequently, the effect of overhydration on arterial stiffness only could be correctly evaluated analyzing the aortic stiffness (carotid-femoral PWV), but not peripheral stiffness. This result has practical importance for noninvasive vascular laboratories evaluation.

In theory, an ECF overload could be the origin of high levels of BP that increase arterial stiffness due to acute vascular passive overdistension (enlargement). This pressure dependent increase of arterial stiffness is determined by the collagen fiber recruitment in response to vascular diameter increases [15]. However, our results showed that the association between ECF/ICF and arterial stiffness is independent of the arterial BP levels. This important finding was evident when arterial stiffness was analyzed using indexes of vascular function that are independent of arterial BP.

It is important to mention that the above-described results include compartment fluid evaluations in absolute (OH) and relative (OH/ECF) indexes, obtained through a widely employed 3-C model. The significant correlation between PWV and indexes associated specifically with overload (OH and OH/ECF) has not been reported previously. Furthermore, we found that arterial stiffness/overhydration status significant relationship should be restricted to the carotid-femoral pathway and not muscular or transitional arteries.

To quantify OH/TBF and mainly OH is important to reach a more integrative evaluation of the hydric status of the patient. These parameters can be obtained from the model published by Chamney et al. in 2007 [17], which stated OH (“MExF”) as a function of ECF, ICF, and weight. A few years ago a novel prediction model was validated, in which body composition and fluid overload (OH) are estimated using a 3-C model. The model uses a correction factor for BMI which was introduced into the body composition monitor (BCM). The 3-C model includes three compartments calculated from ECF and TBF estimations: OH, LTM, and ATM. To this end, constant hydration ratios of the normohydrated LTM and ATM are assumed [17] and the model determines whether changes in ICF and ECF reflect increase or loss of ATM or LTM. OH represents the excess fluid (fluid overload) stored almost exclusively in the extracellular volume of a patient and is therefore part of the ECF, whereas the water of LTM and ATM consists of differing proportion of extracellular and intracellular water in addition to solid components. Healthy individuals are considered to be “normally hydrated” and therefore have virtually no OH. These individuals may be characterized in terms of ATM and LTM only. As the extracellular hydration of LTM and ATM is known, the expected “normal” volume of ECF of these tissues can be calculated. The difference between “normal” ECF and measured ECF is the excess fluid, OH. A negative OH means that the patient is under- or dehydrated. Both in healthy subjects and in chronic kidney disease patients, the distribution of LTM and ATM will lead to significant differences in the ECF/ICF ratio. Therefore ECF/ICF alone does not provide enough information about the hydration status. Therefore, in order to quantify the absolute and/or relative overhydration, other parameters than ECF/ICF are needed.

### 4.1. Methodological Aspects and Limitations

The *β* index used to evaluate the arterial stiffness provided information of the vascular wall state with independence of the BP level. This is not a minor issue since significant improvements of arterial wall stiffness without changes in BP levels have been reported [7].

Aortic PWV was evaluated in all cases using right and left carotid-femoral pathways in order to provide a complete screening of the elastic arteries involved in this vascular territory. According to a recent publication there were some differences between the right and the left carotid-femoral pathways [18]. As shown in the four figures that summarize this research, the above-mentioned differences seem to have minimal relevance in the analyzed population.

A limitation of this study is the lack of data about arterial stiffness before the beginning of renal function replacement therapy. However, as shown, significant association between arterial stiffness and the hydration status could be found.

Finally, as recently commented by Covic et al. [19], despite the extensive use of the modern bioimpedance technique as the bedside technology, there are some potential limitations. Among them, extreme or morbid obesity (BMI ≥ 40) is a significant limitation of the use of bioimpedance [19]. It is important to point out that SH patients included in our study showed lower values of BMI (29.71 ± 5.72 kg/m^2^) and no values of BMI ≥ 40 were observed, with 38,22 kg/m^2^ as the highest observed value.

### 4.2. Clinical Relevance

Elastin is the main constituent of the aortic wall, determining a high level of vascular distensibility. On the other hand, peripheral muscular arteries have lower compliance levels than the aorta. In physiological states, there is an arterial stiffness gradient between the aorta and the peripheral muscular arteries. The mentioned gradient of arterial stiffness determines the existence of reflection sites that dampen the transmission of travelling pressure waves from the aorta to the microcirculation. Additionally, peripheral arterial waves in the diastolic period arrive to the aortic root increasing coronary blood flow. In physiological condition, the aging process determines an aortic stiffness increase that is higher than that observed in muscular peripheral arteries. Consequently, the above-mentioned gradient between elastic and muscular arteries decreases. The reversal of the aortic-peripheral arteries stiffness gradient has been proposed as an index of vascular damage [20, 21]. Recently, Fortier et al. [22] showed that the measurement of the aortic (a central elastic artery) and brachial arteries stiffness (a peripheral muscular artery) and the quantification of the “stiffness gradient” (i.e., aortic-brachial arterial stiffness mismatch) could be useful in adult hemodialyzed patients and has been proposed as a new index and as a prognostic marker. The mentioned authors evaluate arterial stiffness mismatch through the PWV ratio, which is the aortic PWV divided by the upper-arm PWV [22]. The PWV ratio was strongly and independently associated with increased mortality in patients on dialysis [22]. This is why when hemodialyzed patients are evaluated, the inclusion of arterial territories different from the carotid-femoral pathway is important. In theory, if we included the mentioned PWV ratio index, our results would show that overhydration (measured as OH and OH/ECF) or an increased ECF/ICF ratio could be associated with an increased PWV ratio. According to our results, overhydration could lead to the attenuation (or even reversion) of the expected arterial stiffness gradient, promoting end-organ damage. In other words, our work suggests a potential mechanism by which overhydration is associated with aortic stiffness in a different way than that evidenced when peripheral arterial stiffness is analyzed with respect to the hydration status. This is not a minor issue, since coronary blood flow could be modified determining changes in the circulatory system dynamics.

Another clinical connotation derived from our results is suggested if we consider that normalization of fluid overload could not be accompanied by changes in arterial stiffness in the peripheral vessels. Unfortunately, previous studies that have evaluated the association between arterial stiffness and hydration status only evaluated elastic arteries (i.e., carotid artery, aorta), without considering that they cannot respond like the muscular arteries [7, 12]. The different response to renal replacement therapy, in terms of arterial stiffness of both arterial evaluated territories, could be explained by the quantitative variation of the amount of arterial wall constituents observed in brachial and aortic arteries. Previous research has pointed out that changes induced by age on arterial elasticity are not uniform and depend on the vascular territory [23].

Finally, as mentioned, considering that in a 2-C model variations in hydration state may have a strong effect on the prediction of fat-free mass, a recent novel prediction model was validated, in which body composition and net and relative OH are estimated using a 3-C model [17]. As mentioned above, our results show that aortic stiffness is associated with indexes of fluid overload, with independence of arterial BP levels.

## 5. Conclusion

In hemodialysis patients, volume status and overload, expressed as extracellular/intracellular fluid ratio (ECF/ICF), extracellular/Total Body Fluid ratio (ECF/TBF), or overhydration (OH), are associated with central (aortic), but not peripheral (axilo-brachial) arterial stiffness values, independently of BP. Guiding the patients towards this target of normohydration leads to improved aortic but not peripheral arteries function.

## Figures and Tables

**Table 1:** Anthropometric, hemodynamic, and blood characteristics for the entire population and the hydric state-related groups.

	Entire group	Normal Hydration State (NHS)	Overhydration (OH)	Subhydration (SH)	*P* value		
	MV ± SD	MV ± SD	MV ± SD	MV ± SD	NHS versus OH	NHS versus SH	OH versus SH
*n*	65	40	12	13			
Gender (female, %)	30.8%	32.5%	25.0%	30.8%			

Time of hemodialysis (month)	65.35 ± 60.54	71.65 ± 59.07	67.75 ± 73.18	43.77 ± 51.50	**0.850**	**0.134**	**0.350**
Age (years)	58.34 ± 15.93	55.98 ± 17.54	64.75 ± 12.48	59.69 ± 12.25	**0.114**	**0.482**	**0.317**
Height (m)	1.63 ± 0.10	1.63 ± 0.10	1.66 ± 0.09	1.62 ± 0.10	**0.446**	**0.643**	**0.303**
Body weight (kg)	71.76 ± 13.98	72.11 ± 11.74	63.75 ± 10.31	78.07 ± 19.75	**0.031**	**0.189**	**0.035**
BMI (kg/m^2^)	26.93 ± 4.69	27.12 ± 3.91	23.28 ± 3.81	29.71 ± 5.72	**0.004**	**0.071**	**0.003**

Systolic blood pressure (mmHg)	127.97 ± 26.49	133.25 ± 24.89	132.92 ± 25.11	107.15 ± 23.76	**0.968**	**0.002**	**0.015**
Mean blood pressure (mmHg)	92.61 ± 16.40	94.92 ± 14.66	98.19 ± 14.27	80.33 ± 18.39	**0.497**	**0.005**	**0.013**
Diastolic blood pressure (mmHg)	74.92 ± 13.63	75.75 ± 11.98	80.83 ± 12.16	66.92 ± 16.78	**0.205**	**0.042**	**0.027**
Heart rate (beats/min)	87.69 ± 15.04	86.63 ± 14.83	88.42 ± 16.42	90.31 ± 15.23	**0.722**	**0.443**	**0.768**

Hemoglobin (g/dL)	10.54 ± 1.77	10.49 ± 1.70	10.94 ± 2.01	10.34 ± 1.83	**0.445**	**0.782**	**0.440**
Hematocrit (%)	33.69 ± 5.38	33.59 ± 5.40	34.61 ± 5.91	33.16 ± 5.13	**0.575**	**0.805**	**0.519**
Serum albumin (g/dL)	3.99 ± 0.34	4.07 ± 0.30	3.74 ± 0.39	3.97 ± 0.29	**0.004**	**0.332**	**0.104**
Calcium (mg/dL)	8.63 ± 0.44	8.61 ± 0.49	8.63 ± 0.28	8.72 ± 0.42	**0.849**	**0.468**	**0.575**
Phosphates (mg/dL)	4.63 ± 0.86	4.66 ± 0.86	4.41 ± 0.95	4.72 ± 0.83	**0.387**	**0.822**	**0.387**
Parathyroid hormone (pg/mL)	357.60 ± 280.32	425.04 ± 313.01	303.27 ± 192.62	205.45 ± 159.58	**0.210**	**0.019**	**0.179**
Serum urea (mg/dL)	147.88 ± 39.00	147.03 ± 42.43	141.92 ± 38.23	156.00 ± 28.46	**0.710**	**0.481**	**0.304**
Total cholesterol (mg/dL)	175.49 ± 40.20	178.90 ± 39.50	154.75 ± 36.55	184.15 ± 42.10	**0.065**	**0.683**	**0.076**
HDL cholesterol (mg/dL)	40.12 ± 13.07	38.10 ± 12.36	46.33 ± 17.44	40.62 ± 9.17	**0.073**	**0.503**	**0.310**
LDL cholesterol (mg/dL)	101.45 ± 34.87	98.25 ± 35.13	97.33 ± 31.48	115.11 ± 36.19	**0.936**	**0.142**	**0.205**
Total triglycerides (mg/dL)	177.23 ± 98.81	195.96 ± 105.95	128.50 ± 83.96	164.58 ± 73.54	**0.049**	**0.327**	**0.264**

MV: mean value, SD: standard deviation, BMI: body mass index, PWV: pulse wave velocity, and *β*: arterial stiffness index. A *P* < 0.05 was considered statistically significate.

**Table 2:** Hydric status for the entire population and the hydric state-related groups.

	Entire group	Normal Hydration State (NHS)	Overhydration (OH)	Subhydration (SH)	*P* value		
	MV ± SD	MV ± SD	MV ± SD	MV ± SD	NHS versus OH	NHS versus SH	OH versus SH
ECF (L)	16.67 ± 3.03	16.75 ± 3.00	17.63 ± 2.40	15.52 ± 3.47	**0.361**	**0.219**	**0.093**
ICF (L)	17.53 ± 4.24	17.81 ± 4.55	16.21 ± 2.82	17.88 ± 4.36	**0.257**	**0.962**	**0.272**
TBF (L)	34.19 ± 6.88	34.55 ± 7.26	33.86 ± 4.88	33.38 ± 7.66	**0.760**	**0.623**	**0.857**
ECF/EIF	0.97 ± 0.14	0.97 ± 0.14	1.10 ± 0.12	0.88 ± 0.09	**0.003**	**0.029**	**0.000**
ECF/TBF	0.49 ± 0.04	0.49 ± 0.04	0.52 ± 0.03	0.47 ± 0.02	**0.005**	**0.033**	**0.000**
OH (L)	1.42 ± 1.55	1.42 ± 0.70	3.68 ± 1.13	−0.66 ± 0.48	**0.000**	**0.000**	**0.000**
OH/TBF (%)	8.04 ± 8.60	8.32 ± 3.41	20.63 ± 4.49	−4.45 ± 3.30	**0.000**	**0.000**	**0.000**

MV: mean value, SD: standard deviation, and ECF and ICF: extracellular and intracellular fluid volume, respectively. TBF: Total Body Fluid volume. OH: overhydration. A *P* < 0.05 was considered statistically significate.

**Table 3:** Arterial stiffness levels for the entire population and the hydric state-related groups.

	Entire group	Normal Hydration State (NHS)	Overhydration (OH)	Subhydration (SH)	*P* value		
	MV ± SD	MV ± SD	MV ± SD	MV ± SD	NHS versus OH	NHS versus SH	OH versus SH
Carotid-femoral (aortic) stiffness							
Right carotid-femoral PWV (m/s)	10.74 ± 3.25	10.40 ± 3.44	12.84 ± 2.27	9.78 ± 2.69	**0.032**	**0.586**	**0.009**
Left carotid-femoral PWV (m/s)	10.48 ± 3.30	10.08 ± 3.37	12.57 ± 3.38	9.84 ± 2.33	**0.036**	**0.821**	**0.034**
Right carotid-femoral *β*	2.65 ± 1.45	2.44 ± 1.52	3.45 ± 1.03	2.54 ± 1.41	**0.046**	**0.847**	**0.100**
Left carotid-femoral *β*	2.56 ± 1.52	2.31 ± 1.47	3.41 ± 1.52	2.60 ± 1.49	**0.036**	**0.553**	**0.214**

Carotid-brachial stiffness							
Right carotid-brachial PWV (m/s)	7.17 ± 1.38	7.11 ± 1.33	7.65 ± 1.84	7.00 ± 1.20	**0.321**	**0.796**	**0.341**
Left carotid-brachial PWV (m/s)	7.15 ± 1.47	7.19 ± 1.34	7.83 ± 1.97	6.58 ± 1.36	**0.273**	**0.179**	**0.109**
Right carotid-brachial *β*	1.15 ± 0.40	1.08 ± 0.37	1.24 ± 0.43	1.30 ± 0.47	**0.283**	**0.105**	**0.758**
Left carotid-brachial *β*	1.17 ± 0.55	1.14 ± 0.46	1.32 ± 0.66	1.17 ± 0.74	**0.362**	**0.879**	**0.646**

MV: mean value, SD: standard deviation, PWV: pulse wave velocity, *β*: regional arterial stiffness index. A *P* < 0.05 was considered statistical significative.
